# ﻿A new species of torrent catfish, *Liobagrusgeumgangensis* (Teleostei, Siluriformes, Amblycipitidae), from Korea

**DOI:** 10.3897/zookeys.1180.105233

**Published:** 2023-09-26

**Authors:** Su-hwan Kim, Seung-woon Yun, Jong-young Park

**Affiliations:** 1 National Institute of Ecology, Seocheon 33657, Republic of Korea National Institute of Ecology Seocheon Republic of Korea; 2 Department of Biological Sciences, College of Natural Sciences, Jeonbuk National University, Jeonju 54986, Republic of Korea Jeonbuk National University Jeonju Republic of Korea

**Keywords:** Catfishes, cytb, DNA, geographical speciation, key, morphometrics, taxonomy

## Abstract

In a recent survey of populations of the Korean torrent catfish *Liobagrus*, a distinctive species was discovered from the Geum River and its tributaries flowing into the western coast of Korea, and here described as a new species, *L.geumgangensis***sp. nov.** It is distinguishable from other congeners by a combination of the following characters: I, 8 pectoral fin-rays; 52–56 caudal-fin rays; a relatively short occiput to dorsal-fin origin distance (6.9–9.8% SL); a short pelvic-fin insertion to anal-fin origin distance (11.9–17.3% SL); a long dorsal-fin base (10.6–13.5% SL); 8–9 gill rakers; 5–8 serrations on the pectoral fin; the body and fins are dark yellow, the margins of the dorsal, anal, and caudal fins are dark brown, but the outermost rim is faintly yellow. Analysis of the cytb gene also confirmed that *L.geumgangensis* is a monophyletic lineage distinct from other congeners.

## ﻿Introduction

The family Amblycipitidae, which includes four genera (*Liobagrus* Hilgendorf, 1878; *Amblyceps* Blyth, 1858; *Xiurenbagrus* Chen & Lundberg, 1995; *Nahangbagrus* Nguyen & Vo in Nguyen 2005), is restricted to southern and eastern Asia, including Pakistan (*Amblyceps*), and across northern India (*Amblyceps*) through Myanmar, Laos, Cambodia, Thailand to Malaysia (all *Amblyceps*), Vietnam (*Nahangbagrus*), China (*Amblyceps*, *Liobagrus*, *Xiurenbagrus*), Korea (*Liobagrus*), and southern Japan (*Liobagrus*) (Chen and Lundberg 1995; Ng and Kottelat 2000; Kim and Park 2002; Wright and Ng 2008; Park and Kim 2010; Kim et al. 2015; Chae et al. 2019).

Genus *Liobagrus* can be distinguished from the genus *Amblyceps* by its pale fin margins, adipose fin confluence with the caudal fins, rounded or truncated caudal fin (Wright and Ng 2008), and main distribution in eastern Asia. Including the most recently described species, *L.brevispina*, 14 species have been reported from the Yangtze River (Changjiang) in mainland China, five species from Korea, and one species from Japan, for a total of 20 species. *Liobagrus* from the Korean Peninsula have been described with the following characteristics: *L.andersoni* Regan, 1908c, is distinguished by upper and lower jaws of equal length; *L.mediadiposalis* Mori, 1936, by a shorter lower than upper jaw; *L.obesus* Son, Kim & Choo, 1987 by fewer serrations on the pectoral fin; *L.somjinensis* Park & Kim, 2010, by the broad yellowish, vertical, crescent-shaped band in the middle of the caudal fin; and *L.hyeongsanensis* Kim, Kim & Park, 2015 by its small size, with a maximum standard length of 90 mm, and body and fins that are entirely yellowish brown without distinct markings.

The Korean Peninsula is largely divided into western and eastern halves by the Baekdudaegan Mountain Range. Recent studies on freshwater fish from Korea have focused on taxonomic reviews of fish separated by this range: *Koreocobitisrotundicaudata* (West), Wakiya and Mori 1929 vs. *K.naktongensis* (East), Kim et al. 2000; *Coreoleuciscussplendidus* (West), Mori 1935 vs. *C.aeruginos* (East), Song and Bang 2015; *Cobitisnalbanti* (West), Vasil’eva et al. 2016 vs. *C.hankugensis* (East), Kim et al. 2003. Following this logic, it is suspected that subpopulations of *L.mediadiposalis*, which are widely distributed across river drainages from the western (Geum River) to the southeastern (Nakdong River) portions of South Korea, may actually represent distinct species (Son 1987; Kim et al. 2006; Kim 2013). In this study, we investigate the identity of *L.mediaposalis* from the Geum River and confirm its distinctiveness from *L.mediadiposalis* s. str., describing it here as *Liobagrusgeumgangensis*, a new species.

## ﻿Materials and methods

### ﻿Morphological analysis

All samples were collected using a scoop net, fixed in 10% formalin, and preserved in 80% ethanol. Count and measurement procedures were taken following Hubbs and Lagler (1964) and Wright and Ng (2008). Fin spines and soft rays were counted from radiographs and bone staining, and the last two elements of the dorsal and anal fins were counted as one ray. Vertebral counts were also made from soft X-ray radiographs (HA80, HITEX), and the three components associated with the Weberian apparatus were excluded (Chen and Lundberg 1995; Zhao et al. 2004). Gill raker counts were made on the left side on the upper limb of the first arch with an additional 20 specimens to avoid damage to the holotype and paratype specimens (see Comparative material examined). Caudal fin-ray counts include principal caudal-fin rays and dorsal and ventral procurrent caudal-fin rays. Measurements were taken using digital calipers (0.01 mm) and expressed as standard length (**SL**) percentages in Table 1 which included data, followed by the number observed for each count and measurements in parenthesis. All Korean specimens are deposited in the Faculty of Biology at Jeonbuk National University, Jeonju, Korea (**CNUC** herein; current acronym is **JNUC**). Other specimens were used as those deposited in the following institutions: Institute of Zoology, Chinese Academy of Sciences (**CAS**); Institute of Hydrobiology, Chinese Academy of Sciences (**IHB**); National Tsing Hua University (**NTHUB**). Counts and measurements for all paratypes, if different from the holotype, are presented in parentheses after the value for the holotype.

**Table 1.** Morphometric data of *Liobargusgeumgangensis* sp. nov.

Morphometrics	Holotype	Paratype (*N* = 20)		
		range	mean	SD
Standard length (mm)	70.7	57.0–88.3	73.8	9.3
**In percentages of standard length**				
Preocciput length	23.0	19.8–24.2	21.8	1.2
Predorsal length	30.7	27.3–31.5	30.1	1.1
Prepectoral length	21.5	18.8–23.4	21.6	1.1
Prepelvic length	44.7	42.7–47.4	45.6	1.4
Distance from occiput to dorsal-fin origin	9.1	6.9–9.8	8.0	0.7
Distance from pectoral-fin origin to dorsal-fin origin	14.7	14.0–16.0	15.0	0.6
Distance from pectoral-fin origin to pelvic-fin origin	24.6	23.3–30.3	27.1	1.7
Distance from dorsal-fin origin to pelvic- fin origin	22.9	21.6–28.1	25.1	1.7
Distance from pelvic-fin origin to adipose- fin origin	16.5	14.8–21.6	18.0	1.4
Distance from pelvic-fin origin to anal-fin origin	14.8	11.9–17.3	14.5	1.4
Anal-fin base length	18.3	16.4–20.4	18.8	1.0
Dorsal-fin base length	12.1	10.6–13.5	11.8	0.8
Occiput to pectoral-fin origin	12.5	10.9–14.3	12.5	1.0
Interorbital width	8.6	6.2–8.5	7.4	0.6
Body width at pectoral-fin origin	17.4	16.2–19.4	17.7	0.9
Body width under dorsal-fin origin	15.2	13.8–17.2	15.6	1.0
Head width	21.7	18.0–22.0	19.6	1.3
Pectoral spine length	9.7	7.9–10.8	9.8	0.7
Dorsal spine length	8.9	6.9–9.7	8.6	0.7
Caudal-fin length	21.0	16.3–21.3	18.7	1.2
Maxillary-barbel length	23.6	17.8–26.9	22.7	2.4
Nasal-barbel length	18.4	14.1–18.9	16.3	1.6
Outer-mental barbel length	13.4	10.0–16.2	13.2	2.0
Inner-mental barbel length	9.6	7.4–11.2	9.1	1.2

### ﻿DNA analysis

For genomic DNA analysis, a piece of pectoral fin was dissected from the specimen and stored in 100% ethyl alcohol. Next, total DNA was extracted with the genomic DNA Prep Kit for blood and tissue (QUIAGEN Co., USA). The mitochondrial cytochrome b gene was amplified by PCR using the method of Kim et al (2006): forward (F- 5’ ACCACCGTTGTHNTTCAACTA 3’) and reverse primer (R- 5’ TAGAATYYTRGCTTTGGGAG 3’). PCR reactions were completed in a total volume of 50 µl, consisting of 2 µl genomic DNA, 1 µl of the 10 µM forward and reverse primer solutions, 24 µl of Premix Taq (Takara, Japan), and 22 µl of distilled water (Takara, Japan). PCR for all specimens was executed in GeneAtlas G-02 thermocycler (Astec, Japan) with initial denaturing step at 94 °C for 2 min and 30 cycles of 45 s at 94 °C, 1 min at 52 °C, and 1 min at 72 °C. A final extension step at 72 °C for 5 min. The PCR amplicons were visualized on a 2% agarose gel stained with LoadingStar (Dyne, Korea) together with negative controls and Takara 1 kB molecular size ladder for preliminary size determination. The final PCR products were run on an ABI-3730XL sequencer (Applied Biosystems, USA).

Molecular analysis was performed using the cytochrome b (cytb) sequences of seven newly obtained populations of *L.geumgangensis*, and data from 36 other Siluriformes specimens, including the genus *Liobagrus*, which are closely related. Sequence data of species other than *L.geumgangensis* were downloaded from the National Center for Biotechnology Information (NCBI) GenBank (Table 2). After aligning, the obtained genetic information through Clustal Omega (total 1138 bp), Mega X was used to prepare genetic distance and maximum likelihood (ML) trees (with GTR+G+I model). Bootstrap assembling was performed with 1000 replications with rapid options.

**Table 2.** List of mitochondrial cytb sequences newly obtained in this study and downloaded from GenBank with information on collection sites in South Korea and country of origin.

Species	Location of voucher specimens	GenBank Accession No.	Remarks in Fig. 5
*Ameiuruscatus*	USA	MG570433	
*Ameiurusmelas*	China	KT804702	
*Ameiurusnatalis*	USA	MG570406	
*Hemibagrusguttatus*	China	KJ458934	
*Hemibagrusmacropterus*	China	JF834542	
*Hemibagrusnemurus*	Malaysia	KJ573466	
*Hemibagrusspilopterus*	Cambodia	JQ343983	
*Horabagrusbrachysoma*	India	KU870467	
*Ictalurusfurcatus*	USA	KM576102	
*Ictaluruspricei*	Mexico	KJ496298	
*Leiocassiscrassilabris*	China	JX867257	
*Liobagrusandersoni*	South Korea	NC032035	
*Liobagrusanguillicauda*	China	JQ026256	
*Liobagrusgeumgangensis*	South Korea (Cheongyang)	OP980981 (this study)	1
	South Korea (Gongju)	OP980982 (this study)	2
	South Korea (Boeun)	OP980984 (this study)	3
	South Korea (Youngdong)	OP980985 (this study)	4
	South Korea (Muju)	OP980983 (this study)	5
	South Korea (Jinan)	OP980986 (this study)	6
	South Korea (Gosan)	OP945750 (this study)	7
*Liobagrushyeongsanensis*	South Korea	MZ066608	
*Liobagruskingi*	China	KC193779	
*Liobagrusmarginatoides*	China	KC473938	
*Liobagrusmarginatus*	China	NC022923	
*Liobagrusmediadiposalis*	South Korea	KR075136	
	South Korea (Sancheong)	OP980987 (this study)	8
*Liobagrusnigricauda*	China	KC316116	
*Liobagrusobesus*	South Korea	DQ321752	
*Liobagrusreini*	Japan	AP012015	
*Liobagrussomjinensis*	South Korea	MN756661	
*Liobagrusstyani*	China	KX096605	
*Noturustaylori*	USA	KP013089	
*Ompokbimaculatus*	India	KY887474	
*Ompokpabda*	Bangladesh	MK007074	
*Pelteobagruseupogon*	China	KJ001784	
*Pelteobagrusfulvidraco*	China	HM641815	
*Pelteobagrusnitidus*	China	HM746659	
*Silurusasotus*	China	JX087351	
*Siluruslanzhouensis*	China	JF895472	
*Silurusmeridionalis*	China	HM746661	
*Silurusmicrodorsalis*	South Korea	KT350610	

## ﻿Results

### ﻿Amblycipitidae Day, 1873

#### ﻿ Liobagrus geumgangensis sp. nov.

Taxon classificationAnimaliaSiluriformesAmblycipitidae

839B3AA4-7E66-5F81-8E34-3BDC61BFDE21

https://zoobank.org/30082398-3DD5-4F22-B58D-E340E9CF38DE

**Figure 1.**
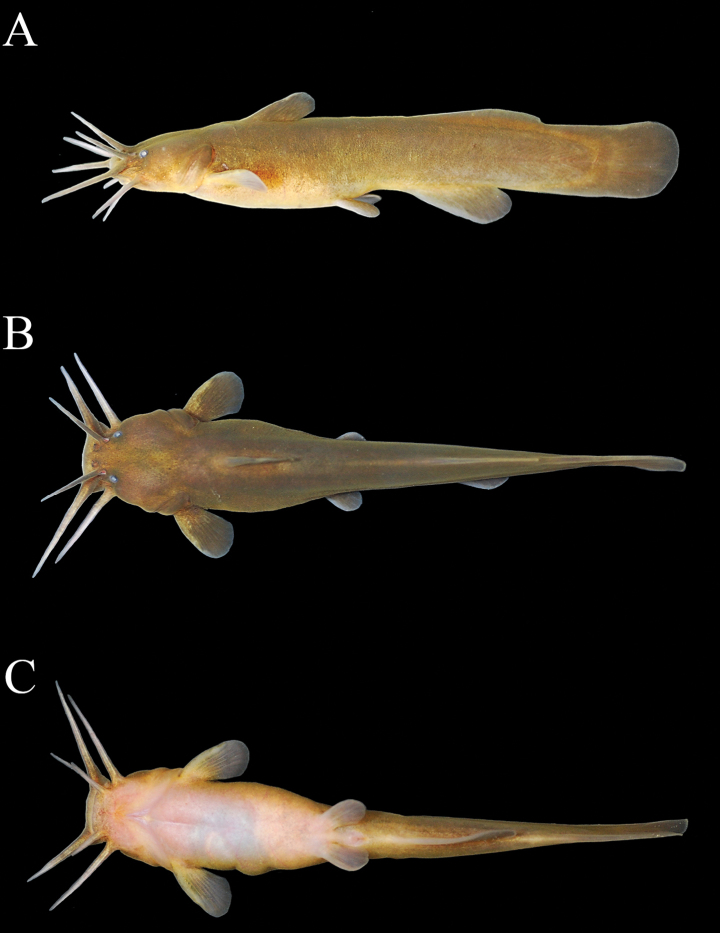
*Liobagrusgeumgangensis* sp. nov. holotype (CNUC 39103), 70.7 mm SL, male, Geum River, Namyang-myeon, Cheongyang-gun, South Korea; lateral (**A**), dorsal (**B**) and ventral (**C**) views.

**Figure 2.**
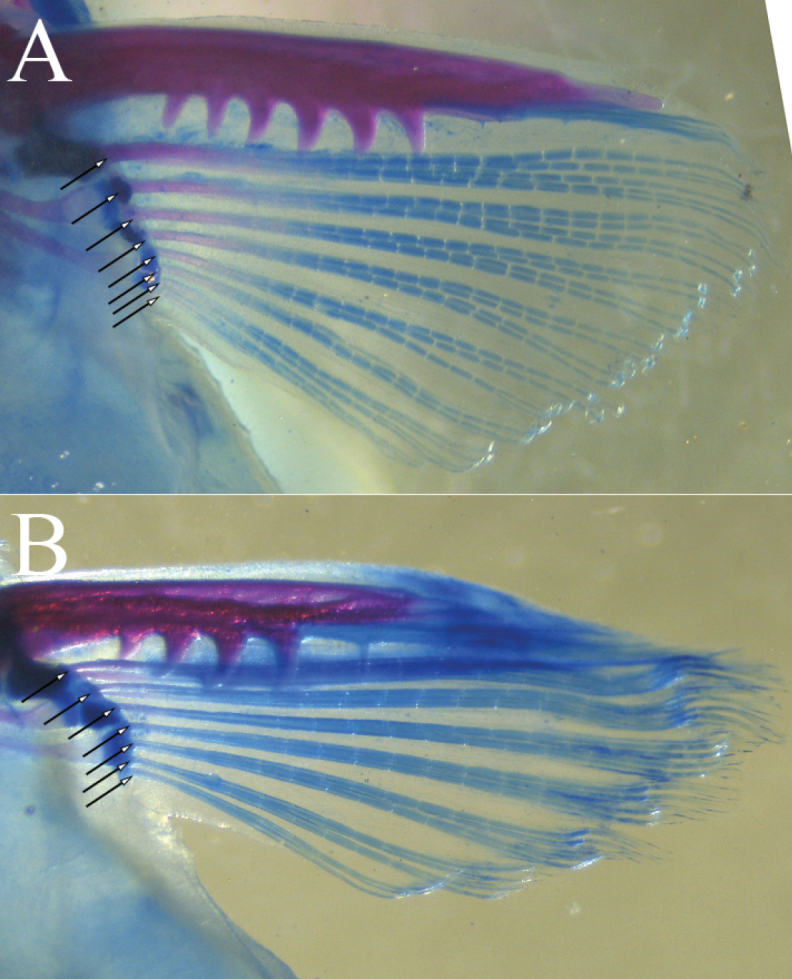
Comparison of pectoral-fin spines and rays of *Liobagrusgeumgangensis* (**A**) and its congener *L.mediadiposalis* (**B**) **A** CNUC 38919, 87.8 mm SL, Geum River, Cheongyang-gun, South Korea **B** CNUC 38930, 77.5 mm SL, Nakdong River, Sancheong-gun, South Korea.

**Figure 3.**
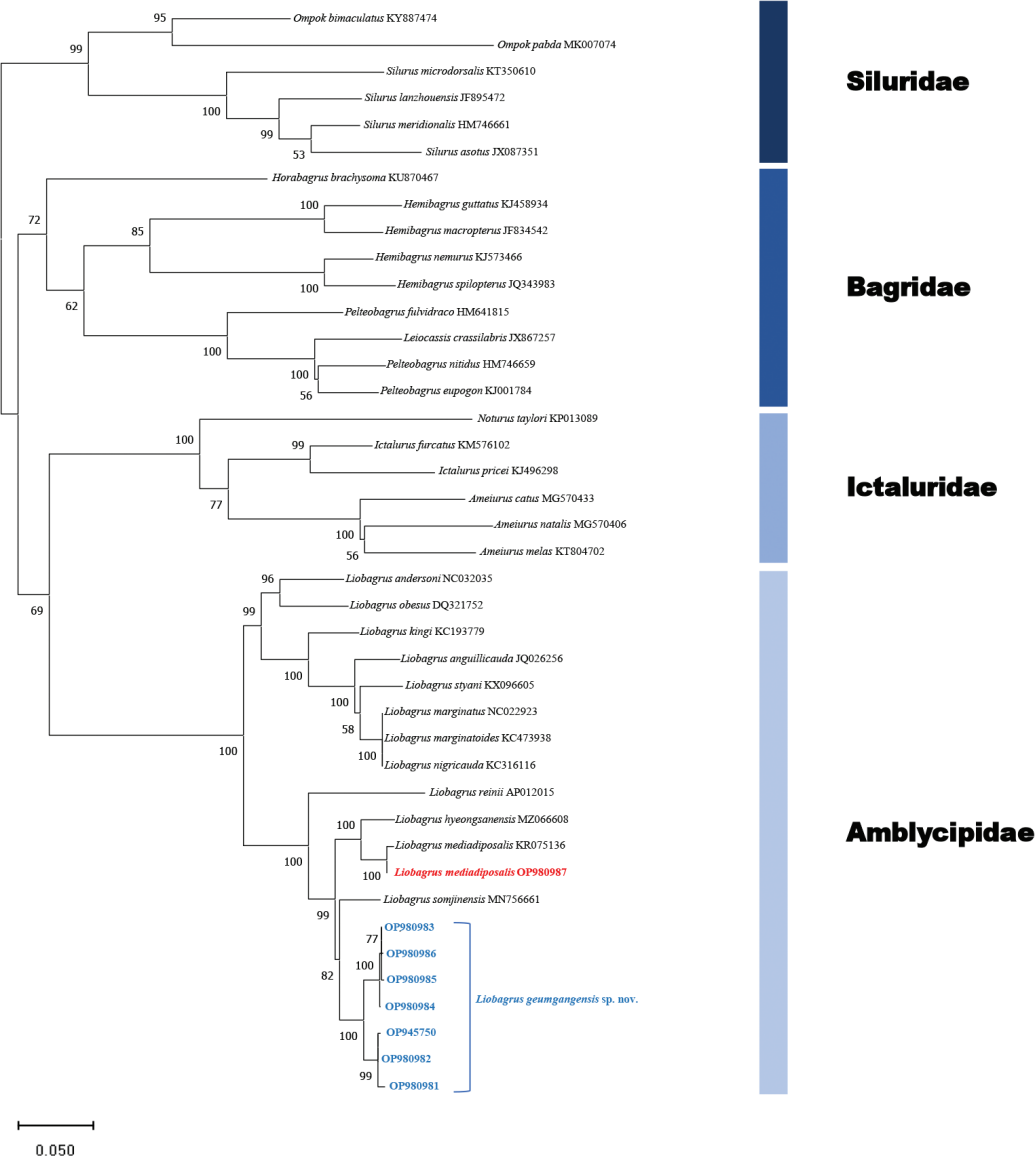
Phylogenetic tree using maximum likelihood (ML) method based on mitochondrial cytochrome b gene sequences of Siluriformes species including *L.geumgangensis*. Bootstrap support values based on 1000 replicates are displayed on each node as >50. The blue color of the branches indicates *L.geumgangensis*.

**Figure 4.**
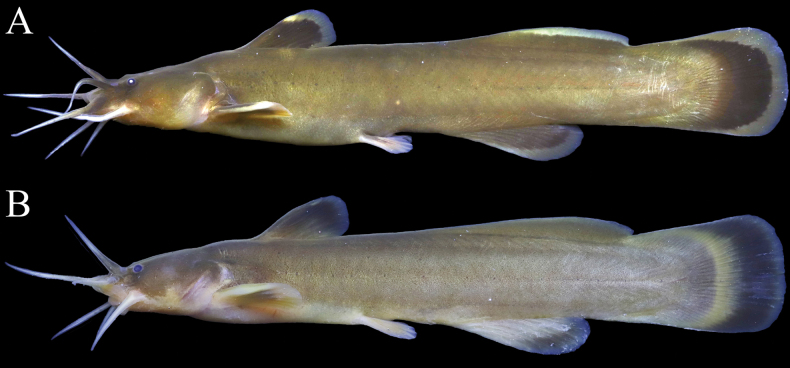
*Liobagrusmediadiposalis* (**A**), CNUC 39153, 97.0 mm SL; male, Nakdong River, Sancheong-gun, South Korea and *L.somjinensis* (**B**), CNUC 39170, 99.4 mm SL, male, Somjin River, Namwon-si, South Korea.

**Figure 5.**
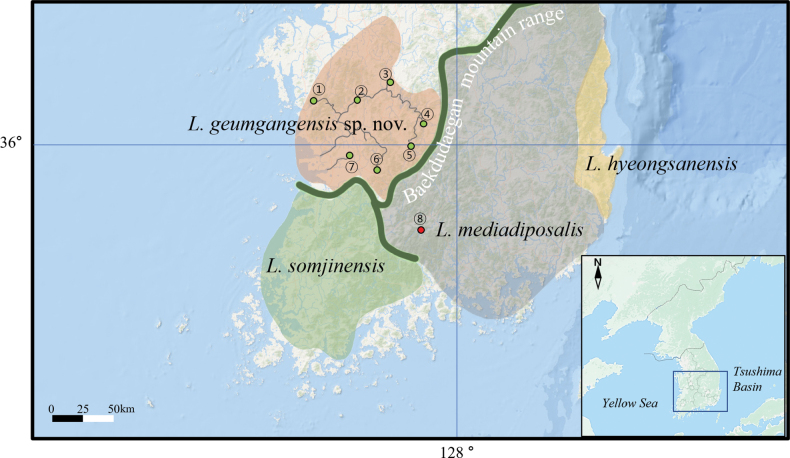
South Korea, showing distributions of *Liobagrusgeumgangensis* (1–7), *L.somjinensis*, *L.hyeongsanensis*, and *L.mediadiposalis*. Dark green line: Baekdudaegan mountain range, showing three biogeographical ranges of ichthyofaunal endemism on the Korean Peninsula (modified from Kim 1997).

Figs 1 , 2 , 3 , 4 , 5 , Tables 1 , 2 , 3

##### Type locality.

Geum River, Korea.

##### Type material.

***Holotype*.** CNUC 39103, 70.7 mm SL, male, Geum River, Namyang-myeon, Cheongyang-gun, South Korea. 36°23'41.66"N, 126°48'41.30"E, collected by J.Y. Park, S.W. Yun and H.T. Kim using a scoop net, 14 March 2018 (Fig. 1). ***Paratypes*.** CNUC 39102, 39104–39113, 39129–39137 (20), 57.0–88.3 mm SL, same data as holotype.

##### Diagnosis.

*Liobagrusgeumgangensis* can be distinguished from other species in the genus by the length of the upper and lower jaws, and the presence or absence of serrations on the posterior edge of the pectoral fin, which are representative characteristics of the genus *Liobagrus*. *Liobagrusgeumgangensis* has a longer upper than lower jaw and serrations on the posterior edge of the pectoral fin. Species that share these characteristics with *L.geumgangensis* are as follows: *L.mediadiposalis*, *L.somjinensis*, *L.hyeongsanensis*, *L.huaiheensis* and *L.pseudostyani*. This new species can be distinguished by the relatively large number of serrations (5–8) on the posterior edge of the pectoral fin (vs. 4–6 in *L.mediadiposalis* and *L.somjinensis*, 2–3 in *L.hyeongsanensis*, *L.huaiheensis* and *L.pseudostyani*). It can be further differentiated from *L.huaiheensis* and *L.pseudostyani* by the subtruncate caudal fin (vs. rounded). *Liobagrusgeumgangensis* is distinguished from its geographically closest congeners *L.mediadiposalis* and *L.somjinensis* by the following combination of characteristics: the body and fins are dark yellow, and the margins of the dorsal, anal, and caudal fins are dark brown, but the outermost rim is faintly yellow (vs. broad yellowish outer margin of the fins in *L.mediadiposalis* and crescent-shaped band in the middle of the caudal fin in *L.somjinensis*); pectoral fin rays I, 8 (vs. both I, 7); caudal fin rays 52–56 (vs. both 57–61); a relatively short occiput to dorsal-fin origin (6.9–9.8% SL vs. 10.3–13.3% in *L.mediadiposalis* and 9.7–13.0 in *L.somjinensis*); a short pelvic-fin origin to anal-fin origin (11.9–17.3% SL vs. 15.3–20.9% in *L.mediadiposalis* and 13.3–18.7% in *L.somjinensis*); a long dorsal-fin base (10.6–13.5% SL vs. 7.7–10.4% in *L.mediadiposalis* and 8.8–11.3% in *L.somjinensis*); and 8–9 gill rakes (vs. 7–11 in *L.mediadiposalis* and 7–9 in *L.somjinensis*) (Figs 1, 2).

##### Description.

Counts and measurements, expressed as percentages of SL, are given in Table 1. Dorsal fin II, 6 (21); origin closer to snout tip than anal-fin origin, posterior margin convex. Dorsal-fin spine equal to or a little shorter than pectoral-fin spine. Adipose fin long, well developed, reaching to or slightly exceeding anal-fin base, confluent with caudal fin, margin convex. Adipose fin depth relatively low. Pectoral fin I, 8 (21); origin anterior to vertical through posterior margin of operculum, partially covered by opercular membrane. Pectoral-fin spine slightly sharp and long, with 4 (4), 5 (8) or 6 (9) serrations on posterior edge. Pelvic fin i, 5 (21); short, located at vertical through, or occasionally slightly anterior to adipose fin; tip not connected to base of anal fin. Anal fin with 14 (1), 15 (2), 16 (9), 17 (7), or 18 (2) rays; distal margin rounded, short, origin placed slightly posterior to vertical through adipose fin origin, posterior tip of anal fin not exceeding posterior margin of adipose fin. Caudal fin subtruncately rounded with 52 (3), 54 (5), 55 (3), or 56 (10). Vertebrae with 38 (9), 39 (8), or 40 (4) post-Weberian elements. Gill rakers 8–9.

Mouth terminal; lips thickened and papillate, premaxillary and mandibular toothpads curved, teeth small and ciliform or setiform, with upper jaw slightly longer than lower jaw. Four pairs of barbels; maxillary barbel longest, reaching to base of pectoral-fin spine; nasal barbel short, not reaching to posterior margin of preoperculum; outer mental barbel long, reaching to posterior edge of pectoral-fin base; inner-mental barbel shortest among four pairs of barbels, about half length of outer mental barbel, reaching to gill membrane. Body compressed and round, head depressed and caudal peduncle strongly compressed; dorsal and ventral profiles straight. Predorsal profile slightly sloping ventrally from dorsal fin to occiput. Eye smaller, dorsolateral, subcutaneous, ovoid. Snout rounded in dorsal view. Anterior nostril tubular, rim with a fleshy flap forming a short tube; posterior nostril porelike, rim posteriorly confluent with base of nasal barbel. Gill membranes narrowly joined at isthmus. Lateral line absent or vestigial.

##### Coloration.

See Fig. 1 for general appearance. In life (Fig. 1): body generally brownish yellow, fading to light yellow on ventral surface, without any distinct markings. All barbels pale gray. All fins with similar color to body, without deep yellow outer margins.

##### Sexual dimorphism.

The adductor mandibulae in males is somewhat swollen during the spawning season, from late April to June.

##### Etymology.

Named after the Geum River (Geumgang in Korean), the type locality. We propose the Korean name Geumgang-Jagasari for this species.

##### Distribution.

*Liobagrusgeumgangensis* sp. nov. is restricted to some rivers flowing to the west coast of South Korea: Geum River and upper stream of Mangyeong River (Fig. 5).

##### Biology and habitat.

*Liobagrusgeumgangensis* is nocturnal and benthic. They inhabit the bottom stratum of large and small stone or pebble substratum in shallow waters, about 0.3–0.6 m deep, with running waters with moderately fast currents. The spawning season is from late April to June. The adult females reach up to 99.8 mm SL and lay eggs 2.5–3.0 mm (2.8±0.1) in diameter. The adult males guard their fertilized egg. They feed mostly on aquatic insect larvae such as those of the Trichoptera, Ephemeroptera and Diptera.

### ﻿Molecular analysis

As a result of the cytb gene analysis, we confirm that samples from *Liobagrusgeumgangensis* form an independent group distinct from the closely related species *L.mediadiposalis* and *L.somjinensis*. Kimura’s 2-parameter distance analysis revealed genetic distances ranging from 0.2% to 2.3% within groups of *L.geumgangensis* populations. We found the genetic distance between *L.geumgangensis* and *L.somjinensis* to be 4.7% to 5.0%, which was smaller compared to that observed with *L.mediadiposalis* (5.8% to 6.1%; Fig. 4). These findings provide robust evidence that *L.geumgangensis* is rather genetically close to *L.somjinensis*, while suggesting that it is a distinctly different species from congeneric species.

## ﻿Discussion

Compared with the original descriptions of *Liobagrus* (Hilgendorf 1878; Gűnther 1892; Regan 1904, 1908a, b, c; Oshima 1919; Nichols 1926; Wu 1930; Tchang 1935; Mori 1936; Son et al. 1987; Wright and Ng 2008; Park and Kim 2010; Kim et al. 2015) and based on coloration and meristic and morphometric data, we confirmed that *L.geumgangensis* sp. nov. is distinct from *L.mediadiposalis*. The number of pectoral fin rays is I, 8 in *L.geumgangensis* (vs. I, 7 in *L.mediadiposalis*) and the number of caudal fin rays is 52–56 (average 54.9) (vs. 57–61). The distance between the occiput and dorsal-fin origin is shorter at 6.9–9.8% SL than *L.mediadiposalis* (10.3–13.3% SL). The relative lengths of the upper and lower jaws are a key characteristic used in identifying *Liobagrus* species, with three possible states identified: lower jaw longer than upper (*L.marginatoides*, *L.marginatus*, *L.kingi*, and *L.chengduensis*), jaws of equal length (*L.andersoni*, *L.obesus*, *L.nigricauda*, *L.anguillicauda*, *L.brevispina*, *L.aequilabris*, and *L.formosanus*), and upper jaw longer than lower (all remaining congeners; Table 3). On the Korean Peninsula, *L.andersoni* and *L.obesus* have jaws of equal length, whereas *L.geumgangensis*, along with *L.mediadiposalis*, *L.somjinensis* and *L.hyeongsanensis*, have a lower jaw shorter than its upper jaw. *Liobagrusmediaposalis*, *L.somjinensis* and *L.hyeongsanensis* all have I, 7 pectoral-fin rays; just two species, *L.andersoni* and *L.geumgangensis* have I, 8 pectoral-fin rays. The pectoral fin rays are not used as a common diagnostic characteristic and are considered to be species-specific variables, but in contrast, some studies have suggested fixed results (Xie et al. 2022). Since this study did not measure a large number (*N* = 30) of *L.andersoni* specimens, it is possible that the number of pectoral fin rays may vary, but this study intends to use this as a comparative characteristic.

**Table 3.** Comparisons of major diagnostic characters of *Liobagrus* species distributed in Korea and neighboring countries.

**Characte**r	***L.geumgangensis***	***L.mediadiposalis***	***L.andersoni***	***L.obesus***	***L.somjinensis***	***L.hyeongsanensis***	***L.reini***	***L.formosanus***	***L.nigricauda***	***L.marginatoides***
Upper/lower jaw in length	>1	>1	=1	=1	>1	>1	>1	=1	=1	<1
Caudal fin shape	Subtruncate	Subtruncate	Subtruncate	Subtruncate	Subtruncate	Subtruncate	Rounded	Rounded	Rounded	Subtruncate
Number of serrations on the pectoral fin	5–8	4–6	0–3	3–5	4–6	2–3	0	0	0	0
Occiput to dorsal-fin origin	6.9–9.8	10.3–13.3	8.5–12.4	9.4–15.1	9.7–13.0	9.3–12.9	8.3–9.4	7.2–10.2	7.6–13.3	8.8–11.6
Pelvic-fin origin to anal-fin origin	11.9–17.3	15.3–20.9	11.9–17.0	9.6–14.1	13.3–18.7	12.1–16.6	12.7–15.3	13.5–19.1	12.1–16.4	13.6–17.9
Dorsal-fin base length	10.6–13.5	7.7–10.4	10.3–15.2	12.4–16.0	8.8–11.3	7.8–10.9	10.6–12.7	11.0–14.4	6.3–12.0	11.0–14.4
**Character**	***L.styani***	***L.marginatus***	***L.anguillicauda***	***L.aequilabris* ^a^**	***L.chenghaiensis* ^b^**	***L.kingi* ^c^**	***L.huaiheensis* ^d^**	***L.chengduensis* ^e^**	***L.pseudostyani* ^e^**	***L.brevispina* ^f^**
Upper/lower jaw in length	>1	<1	=1	=1	≤ 1	≤ 1	>1	<1	>1	=1
Caudal fin shape	Rounded	Subtruncate	Rounded	Rounded	Subtruncate	Rounded	Rounded	Rounded	Rounded	Rounded
Number of serrations on the pectoral fin	0	3–4	0	0	2–5	2–4	2–3	3–4	2–3	0
Occiput to dorsal-fin origin	5.8–11.4	8.8–12.1	8.0–10.7	–	–	–	–	–	–	–
Pelvic-fin origin to anal-fin origin	10.3–14.1	12.3–17.2	12.0–14.6	–	–	–	–	–	–	–
Dorsal-fin base length	8.2–12.7	10.8–15.3	8.5–10.7	–	–	–	–	–	–	–

a, Wright and Ng 2008; b, Sun et al. 2013; c, Xie and Zhang 2018; d, Chen et al. 2021; e, Chen et al. 2022; f, Xie et al. 2022.

To summarize, within the *Liobagrus* distribution in Korea, it is noteworthy that *L.geumgangensis* is the only species with an upper jaw longer than the lower jaw and I, 8 pectoral fin rays. In addition, *L.geumgangensis* differs from *L.somjinensis* in several characters: absence vs. presence of a broad vertical yellowish crescent-shaped band on the caudal fin, with a deep black outer margin; 5–8 vs. 4–6 serrations on the pectoral fin; and 52–56 caudal fin rays vs. 57–61 (Fig. 4).

*Liobagrusgeumgangensis* is allopatrically distributed in restricted river systems, the Geum River and a patch of the Mangyeong River, which flow toward the western coast of Korea. Unlike other *Liobagrus* species, *L.somjinensis* is restricted to rivers flowing to the west coast and Geogeum Island, the Somjin, Dongin and Tamjin rivers. *Liobagrushyeongsanensis* has a much smaller distribution including the Hyeongsan River flowing into the east coast, whereas *L.mediadiposalis* is found only in the Nakdong River on the west coast (Fig. 5). *Liobagrusandersoni* is restricted to the Han River system alone. Meanwhile, *L.obesus* has a wide distribution including the western river systems of Geum, Yeongsan and Mangyeong, and their distribution overlaps with other congeners. Interestingly, based on morphological and genetic research, all six species of the Korean *Liobagrus* are endemic (Kim et al. 2006, 2015; Park and Kim 2010; Kim 2013).

Our results are consistent with a recent study by Kim et al. (2006) on the evolutionary relationships among *L.mediadiposalis* populations in Korea using cytochrome b sequences. Kim et al. (2006) considered Type I to be the Somjin River group, which included four populations (Dongin, Somjin, and Yeongsan rivers and Geogeum Island), and Type II as the Geum River group comprising a single population. Although they confirmed that the Geum River group was genetically distinct from *L.mediadiposalis*, the taxonomy of this population was not further investigated. Here, our analysis of seven different populations confirms that the Geum River group represents a distinct, unnamed species (Fig. 3).

The geographical isolation of the freshwater fish of South Korea is likely related to the geological origins of the Korean Peninsula. The Korean Peninsula was formed by active orogeny movements from the early Triassic to the Jurassic. The structure of the current complex topography is due to the tectonic shift concentrated in the Tertiary Period of the Cenozoic Era. As a result, Korea has an approximately 1400 km long mountainous region stretching from North Korea to South Korea. The peninsula, called the Baekdudaegan, contains at least 12 mountain ranges that have formed unique rivers, including the Han, Geum, Nakdong, Hyeongsan, and Mangyeong rivers (Chough et al. 2000; Sohn et al. 2001). Consequently, some Korean freshwater fish are distributed allopatrically, causing speciation.

According to Mori (1936) and Kim (1997), the freshwater fish fauna on the Korean Peninsula is divided into three biogeographical regions: northeast, west, and southern Korea subdistricts. Such biogeographical distributions based on large mountain ranges creating physical barriers and allopatric distributions lead to endemic species (Fig. 5). In particular, Korean cobitid fishes, including 10 endemic species, are the best example of a diverse regional distribution (Kim and Park 2002; Chae et al. 2019). Some mountain ranges between the west and south subdistricts serve as strong long-term barriers to recurrent gene flow. Such barriers led to the split of *Coreoleuciscussplendidus* populations into two genetically discrete subdistrict populations (Kim et al. 2012). Geographical speciation has also been observed among some species of the Cyprinidae family on the Korean Peninsula (Kim 1997; Kim and Park 2002; Park and Kim 2010). Isolation of fish populations in different river systems has led to high levels of endemism in Korean *Liobagrus*, including *L.geumgangensis*.

### ﻿Key to species of *Liobagrus* distributed in Korea

**Table:** 

1	The length of both jaws is the same, and the mouth is in front of the snout	**2**
–	The length of both jaws is different, the lower jaw is shorter than the upper jaw, and the mouth is located below the snout	**3**
2	The pectoral fin spines have 0–3, and the adults have small and traces	***L.andersoni***
–	The spines of the pectoral fin are 3–5, and the spines of the adult are large and clear	***L.obesus***
3	There are no bright bands on the edges of the dorsal fin, anal fin, caudal fin, and adipose fins, while a crescent-shaped bright band appears in the center of the caudal fin	***L.somjinensis***
–	The dorsal fin, anal fin, caudal fin, and adipose fin have bright colored bands, while there is no crescent-shaped bright band in the center of the caudal fin	**4**
4	The edges of the anal fin, caudal fin, and adipose fin have a bright band. The pectoral fin spines are long (more than 7% of the standard length), and the dorsal fin spines are also long (more than 5% of the standard length)	**5**
–	There are narrow or almost no light-colored bands along the edges of the anal, caudal and adipose fins. The length of the pectoral fin spines is short (less than 6% of the standard length), and the length of the dorsal fin spines is also short (more than 4% of the standard length)	***L.hyeongsanensis***
5	A wide band of bright color appears on the edge of each fin, the number of rays of the pectoral fin is I, 7, and the distance between the origin of the pelvic fin and the origin of the anal fin is long (more than 17% of the standard length)	***L.mediadiposalis***
–	There is a bright band at the edge of each fin, but the width is narrow. The number of rays of the pectoral fin is I, 8, and the distance between the origin of the pelvic fin and the origin of the anal fin is short (less than 15% of standard length)	***L.geumgangensis* sp. nov.**

### ﻿Comparative material examined

*Liobagrusandersoni*: CNUC 9441–3, 3 specimens, 86.3–90.9 mm SL, Bongpyeong-myeon, Pyeongchang-gun, Gangwon-do, Han River, South Korea; CNUC 39021, 1 specimen, 71.5 mm SL, Cheongchen-myeon, Goesan-gun, Chungcheongbuk-do, Han River, South Korea. CNUC 38753–38778, 26 specimens, 81.4–105.5 mm SL, Gasoo-ri, Jeongseon-eup, Jeongseon-gun, Gangwon-do, Han River, South Korea.

*Liobagrusobesus*: CNUC 936–40, 5 specimens, 58.2–81.8 mm SL, Simcheon-myeon, Yeongdong-gun, Chungcheongbuk-do, Geum River, South Korea: CNUC 9434, 9436, 9439, 3 specimens, 82.3–94.8 mm SL, Simcheon-myeon, Yeongdong-gun, Chungcheongbuk-do, Geum River, South Korea: CNUC 39022, 1 specimen, 95.5 mm SL, Gosan-myeon, Gosan-gun, Jeollabuk-do, Mankyeong River, South Korea.

*Liobagrusmediadiposalis*: CNUC 37821–38, 18 specimens, 73.9–100.3 mm SL, Sicheon-myeon, Sancheong-gun, Gyeongsangnam-do, Nakdong River, South Korea: CNUC 1019–20, 2 specimens, 55.6–64.7 mm SL, Imgok-dong, Gwangsan-gu, Gwangju, Yeongsan River, South Korea: CNUC 1025–8, 4 specimens, 78.9–108.9 mm SL, Hamyang-gun, Gyeongsangnam-do, Nakdong River, South Korea: CNUC 1035–8, 4 specimens, 82.4–109.5 mm SL, Yurim-myeon, Hamyang-gun, Gyeongsangnam-do, Nakdong River, South Korea: CNUC 1801–5, 5 specimens, 83.4–107.8 mm SL, Macheon-myeon, Hamyang-gun, Gyeongsangnam-do, Nakdong River, South Korea: CNUC 1806–8, 3 specimens, 81.9–102.6 mm SL, Cheoncheon-myeon, Jangsu-gun, Jeollabuk-do, Geum River, South Korea: CNUC 1809, 1 specimen, 89.4 mm SL, Samcheok-si, Gangwon-do, Samcheokoship Stream, South Korea: CNUC 9445, 1 specimen, 123.7 mm SL, Mungyeong-eup, Mungyeong-si, Gyeongsangbuk-do, Nakdong River, South Korea: CNUC 39153, 1 specimen, 97.0 mm SL, Sicheon-myeon, Sancheong-gun, Gyeongsangnam-do, Nakdong River, South Korea.

*Liobagrussomjinensis*: CNUC 37749, 1 specimen, 99.4 mm SL, Geumji-myeon, Namwon-si, Jeollabuk-do, Somjin River, South Korea: 37750–65, 16 specimens, 74.1–100.6 mm SL, Geumji-myeon, Namwon-si, Jeollabuk-do, Somjin River, South Korea: CNUC 37766–9, 4 specimens, 74.8–91.3 mm SL, Jukgok-myon, Gokseong-gun, Jeollanam-do, Somjin River, South Korea: CNUC 39170, 1 specimen, 99.4 mm SL, Sikjeong-dong, Namwon-si, Jeollanam-do, Somjin River, South Korea.

*L.hyeongsanensis*: CNUC 38547, 1 specimen, 84.1 mm SL, Yangbuk-myeon, Gyeongju-si, Hyeongsan River, South Korea: CNUC 38548–38567, 20 specimens, 57.1–84.1 mm SL, Yangbuk-myeon, Gyeongju-si, Hyeongsan River, South Korea: CNUC 38830–38854, 25 specimens, 41.8–67.8 mm SL, Hwangnyong-dong, Gyeongju-si, Bukcheon River, South Korea.

*L.geumgangensis* (for gill raker analysis): CNUC 39138–39142, 39170–39184, 20 specimens, 60.2–95.7 mm SL, Namyang-myeon, Cheongyang-gun, Chungcheongbuk-do, Geum River, South Korea.

*L.reini*: CNUC 38971–38972, 2 specimens, 48.9–64.5 mm SL, Sanda, Hyogo Prefecture, Japan.

*L.formosanus*: NTHUB 01763, 7 specimens, 56.6–86.4 mm SL, Puli, Nantou County, Taiwan; NTHUB 01766, 1 specimen, 73.3 mm SL, Changhua County, Taiwan.

*L.nigricauda*: IHB 0110, IHB 0114, IHB 0366, IHB 0379, IHB 83135, IHB 920503, IHB 8840595, IHB 8841429, 13 specimens, 54.5–94.2 mm SL, Sichuan Province, China.

*L.marginatoides*: CAS 5224, CAS 5227, CAS 5230, CAS 5234, CAS 5332, 5 specimens, 51.8–58.6 mm SL, Sichuan Province, China.

*L.styani*: CAS 770020–4, 5 specimens, 52.0–95.3 mm SL, Shanxi Province, China; CAS 80-1317, CAS 80-0897, CAS 80-1204–5, CAS 90-0205, CAS 90-0207, CAS 90-0209, CAS 81-1371, CAS 81-1373, 9 specimens, 67.5–101.5 mm SL, Jangxi Province, China.

*L.marginatus*: CAS 177637–177684, 48 specimens, 83.1–116.2 mm SL, Sichuan Province, China.

*L.anguillicauda*: CAS 131004–131039, 36 specimens, 87.9–98.6 mm SL, Anhui Province, China.

## Supplementary Material

XML Treatment for Liobagrus geumgangensis

## ﻿Additional information

### Author ORCIDs

Su-hwan Kim https://orcid.org/0000-0003-4831-5071

Seung-woon Yun https://orcid.org/0000-0003-0893-4468

Jong-young Park https://orcid.org/0000-0003-2348-5111

### Data availability

All of the data that support the findings of this study are available in the main text.
